# Borders and Businesses: Intersectional Resilience, Entrepreneurship and Health Promotion Among Immigrant Women in the Post-Pandemic Global South

**DOI:** 10.3390/ijerph23030324

**Published:** 2026-03-05

**Authors:** Heila Magali da Silva Veiga, Aleida Mendes Borges, Kamila Batista de Melo, Heloisa Carlos Reis, Nayara Zanata

**Affiliations:** 1 Campus Umuarama, Universidade Federal de Uberlândia, Uberlândia 38405-318, MG, Brazil; kamilabmelo@gmail.com (K.B.d.M.); heloisacarlosreis2@gmail.com (H.C.R.); nayaramzanata@gmail.com (N.Z.); 2 King’s College London, London WC2R 2LS, UK; aleida.c.mendes_borges@kcl.ac.uk

**Keywords:** immigrant women entrepreneurship, intersectionality, Global South, public policies, women’s health, social determinants of health, mental health, social support

## Abstract

**Highlights:**

**Public health relevance—How does this work relate to a public health issue?**

- Women’s entrepreneurship in vulnerable contexts is directly linked to mental health, stress exposure, and psychosocial well-being in the Global South.
- Economic exclusion, informality, and lack of social protection act as structural social determinants of health for immigrant women.

**Public health significance—Why is this work of significance to public health?**

- Integrating entrepreneurship support with mental health and social policies can reduce stress-related health risks and promote resilience among vulnerable women.
- The paper highlights how intersectional inequalities amplify health disparities during and after public health crises.

**Public health significance—Why is this work of significance to public health?**

- Public health policies should incorporate mental health, social support, and collective strategies into entrepreneurship and labor inclusion programs.
- Health promotion strategies targeting immigrant and vulnerable women must address economic, social, and psychological dimensions simultaneously.

**Abstract:**

This theoretical paper examines immigrant women’s entrepreneurship in the post-pandemic Global South. We challenge conventional models that fail to capture economic engagement driven by necessity and survival. Using an intersectional lens, the analysis illuminates how structural vulnerabilities such as—gender, class, income, migratory status, and social hostility—hinder formal market integration. COVID-19 related restrictions exacerbated this precarity, disproportionately affecting informal businesses. We contend that entrepreneurship serves as a pivotal strategy for socio-economic resilience and our findings advocate for holistic public policies that transcend microcredit by integrating psychosocial support, language acquisition, and anti-discrimination measures. Furthermore, we underscore the role of collective strategies and community networks in ensuring business sustainability and shifting the focus toward systemic inclusion. By evidencing how entrepreneurship, when embedded in supportive policies and collective networks, contributes to mental health protection, stress reduction, and psychosocial well-being, this paper highlights immigrant women’s entrepreneurship as a relevant strategy for health promotion and social resilience in the Global South.

## 1. Introduction

The growth of entrepreneurship in the global landscape has been widely celebrated as a driver of economic development, innovation, and productive inclusion [1] However, this dominant narrative, largely influenced by concepts originating in the Global North, is based on assumptions that do not necessarily apply the realities of the Global South, characterized by historical inequalities, economic instability, and institutional fragility [2,3,4]. By privileging indicators of growth and competitiveness, these assumptions neglect the structural conditions shaping the entrepreneurial trajectories of low-income women, for whom entrepreneurship is often not a strategic choice, but an urgent necessity in the face of exclusion from formal labor market [5]. As such, it undermines the impact of multiple social markers, such as gender, race/ethnicity, class/income, and migratory status, producing additional layers of inequality [6,7] to the experience of low-income women across Global South.

Drawing on intersectionality as a critical social theory concerned with the analysis of power relations and structural inequalities, which goes beyond the analysis of coexisting social markers [8], this paper examines how these dimensions are mutually constitutive and embedded in systems of domination and resistance. From this perspective, social inequalities are produced through intersecting domains of power that operate at structural, institutional, cultural, and interpersonal levels, shaping access to resources, social protection, and well-being. This approach is particularly relevant for understanding the value of entrepreneurship for low-income women, as it allows the analysis to move beyond individual characteristics and focus on how historical, political, and social arrangements condition economic participation and health-related outcomes.

Furthermore, by centering the matrix on domination, intersectionality illuminates how overlapping axes of marginalization dictate the strategies of resilience adopted by women in the Global South [9]. Across Latin American, African and Asian countries [1,3,10] intersectionality for low-income women entrepreneurs manifests particularly in persistent barriers such as restricted access to credit, the absence of institutional support, and structural discrimination. This was further exacerbated during the implementation of COVID-19 related restrictions exposing the fragility of social protection systems [11,12,13], with the consequent contraction of demand and the intensification of domestic burdens disproportionately affecting women [14], and forcing many to adopt emergency survival strategies through informal work and the expansion of community support networks [15,16]. To avoid reducing economic inclusion to mere formalization or market participation, it is essential to define the concept explicitly from the outset. In this paper, economic inclusion is understood as sustained and non-discriminatory access to productive opportunities, social protection, labour rights, institutional networks, and financial resources under conditions that ensure dignity, autonomy, and medium- to long-term stability. In contrast to conventional notions of “performance”, typically centred on growth rates, profitability, or scalability, the conception adopted here incorporates social and psychosocial dimensions. Entrepreneurial resilience, therefore, is not framed simply as individual adaptation to crisis, but as the capacity to sustain viable economic trajectories within settings shaped by entrenched inequalities and institutional constraints [5,15]. In this context, female entrepreneurship functions primarily as a form of socio-economic resilience, guided by subsistence and social cohesion rather than solely by profit [9,17].

Against this backdrop of increased vulnerability, public policies become key instruments for change. However, a critical analysis of state-led capacity building initiatives reveals a pattern of structural oversight: while successful in creating pathways for formalization and financial inclusion through technical means (e.g., Brazil’s MEI scheme, microfinance in Mexico, or gender-responsive procurement in Nigeria and Ethiopia [1,18,19], these programs show a pronounced bias towards technical "hard skills" and economic metrics. The digital dimension also deserves attention in support strategies. While the adoption of digital technologies accelerated during the pandemic not all entrepreneurs had access to the tools and training needed to use them effectively. This is crucial because Public policies and collective initiatives that incorporate digital inclusion not only expand markets, but also strengthen autonomy and the capacity to adapt to new crises. Thus, coordinated action between state agencies and civil society actors is decisive to ensure that there is knowledge bridging and appropriate programs supporting such transitions [9,20].

Crucially, an important dimension frequently overlooked by current frameworks is the indispensability to integrate mental health and social support to address psychological well-being as key to the effectiveness of such programs. This is critical because the immense pressure to ensure family survival, combined with the overlap of productive and reproductive roles, generates high levels of stress and psychological strain. Research in Kenya and Nigeria, for example, revealed that pandemic-induced psychological strain and time poverty forced women to reduce their time devoted to paid employment by between 29% and 46%, directly undermining economic sustainability [10,11,21].

From a public health perspective, these limitations reflect a broader inattention the political and social determinants of health as these outcomes are not solely contingent on individual behaviors or economic conditions, but are fundamentally mediated by policy decisions, institutional arrangements, and asymmetric power relations that structure access to resources, protection, and care. Dawes (2020) [22] conceptualizes these dynamics as political determinants of health, theorizing how governance, policy design, and state priorities actively engender health inequities. In alignment with this view, the equitable aging in health framework proposed by Perone et al. (2025) [23] adopts a multilevel approach, illustrating how structural, institutional, and social domains of power interact longitudinally throughout the life course to shape vulnerability and resilience. Together, these perspectives underscore the need to conceptualize women’s entrepreneurship not merely as an economic activity, but as a socio-political phenomenon embedded in intersecting economic, political, social, and health-related structures.

This paper adopts a theoretical approach grounded in the analysis of interdisciplinary scholarship on women’s entrepreneurship, intersectionality, public health, and social policy. It focuses on three core dimensions: (1) structural vulnerability and intersecting inequalities affecting immigrant women; (2) entrepreneurship as a survival-oriented and resilience-building strategy; and (3) the role of public policies, social support, and collective strategies in shaping health and well-being. Priority was given to peer-reviewed papers and institutional reports (gray literature) addressing the Global South and migration contexts, particularly in post-pandemic settings. The discussion is structured thematically, moving from structural conditions to collective responses and policy implications, in order to connect individual entrepreneurial experiences with broader social and health-related processes.

Therefore, this theoretical paper critically analyzes the entrepreneurship of low-income women in the post-pandemic context, articulating the intersecting dimensions that shape their experiences while addressing the limitations of policy frameworks that prioritize economic metrics.By questioning conventional entrepreneurship models, the paper proposes shifting the focus from isolated economic performance to a holistic approach that incorporates soft-skill development, robust support networks, and accessible mental health resources.Ultimately, it argues that effective support for women entrepreneurs in the Global South must adopt an intersectional and integrated approach capable of fostering sustainable growth, resilience, mental health, and social inclusion. From a public health perspective, strengthening women’s entrepreneurial ecosystems through integrated policies addressing mental health, social protection, and collective support operates as a health-promotion strategy that mitigates stress-related outcomes, psychosocial suffering, and long-term health inequities.In doing so, the paper seeks to bridge the gap between entrepreneurship and public health. Moving beyond scholarship that treats women’s entrepreneurship and mental health as loosely connected, the paper explicitly frames entrepreneurial activity as a socially embedded pathway to health promotion and resilience. By foregrounding the interaction between power structures and policy environments, the paper offers a rigorous analysis of how economic agency shapes psychosocial well-being in the post-pandemic Global South.

## 2. Classical Conceptions of Entrepreneurship and Their Limitations in the Global South

The dominant understanding of entrepreneurship, shaped by authors such as Schumpeter (1982) [24], conceives the entrepreneur as an agent of innovation, endowed with technical and managerial skills, capable of identifying opportunities and transforming them into scalable economic outcomes. Although this definition has guided business policies and practices for decades, it reflects a specific frame of reference: one rooted in contexts of relative capital abundance, institutional stability, and developed market characteristics typically associated with the Global North [2,3]. When transposed to countries in the Global South, contradictions emerge, revealing its inability to capture the complexity of the entrepreneurial trajectories of vulnerable populations [25,26].

The emphasis on individual autonomy and competitiveness hallmarks of the Schumpeterian vision and its neoliberal derivatives obscures entrepreneurial practices grounded in cooperation, reciprocity, and survival [2,13]. For low-income women, entrepreneurship rarely comes from a rational calculation aimed at maximizing profit; rather, it is often the result of exclusion from the formal labor market, the absence of employment policies, and the immediate need to maintain one’s household [13]. As such, the traditional lens fails to explain phenomena that do not align with growth metrics but have significant social and community relevance.

The myth of the “natural entrepreneur” also neglects the centrality of structural inequalities that shape access to resources and opportunities [11]. In the Global South, the precariousness of physical infrastructure, political instability, and the scarcity of formal credit are barriers that, combined with gender and racial prejudice in some contexts, shape the scope of women entrepreneurs’ activities [2]. The critical literature argues that such barriers are not incidental but structural and that ignoring them perpetuates exclusion under the guise of “entrepreneurial meritocracy” [3].

Over the past decades, there have been attempts to adapt the concept of entrepreneurship to peripheral realities, through approaches such as social entrepreneurship, inclusive entrepreneurship, and immigrant entrepreneurship [27,28]. However, even these formats, while advancing towards the recognition of social impacts, still carry traces of the dominant economic paradigm, prioritizing efficiency and competitiveness over dimensions such as well-being, safety, and community cohesion [13,18]. Adaptation without epistemological rupture risks importing models that are alien to local needs.

The entrepreneurship of low-income women in the Global South displays characteristics that directly challenge the foundations of the traditional model. The hybrid nature of their activities that combine production, service provision, caregiving, and community mobilization contests established analytical categories that compartmentalize work into productive and reproductive spheres [26]. This duality is rendered invisible by conventional metrics but is central to understanding women’s resilience and adaptability in contexts of vulnerability.

In Brazil, data from the Overview of Female Entrepreneurship [29] indicate that more than 10 million women manage businesses, mostly informal, concentrated in services and commerce, with recurrent barriers to credit and training. In Latin America, UN Women (2024) [30] highlight that entrepreneurs remain disproportionately represented in subsistence activities, while in African contexts, studies show that microfinance and self-help groups are essential mechanisms of resilience for women facing systemic exclusion [31]. These regional patterns reinforce that women’s entrepreneurship in the Global South is often characterized by necessity rather than strategic choice, leading to the prevalence of subsistence activities over high-growth ventures. Data consistently point to a systemic failure to remove institutional barriers, including restrictive collateral requirements, limited access to formal networks, and biases in technical training programs. Similar patterns are observed across Latin America, where institutional reports highlight persistent barriers to credit, training, and formalization for women entrepreneurs [32]. This structural exclusion forces reliance on informal mechanisms, where collective resilience—though vital for survival—cannot act as a substitute for the strategic, sustained support that only comprehensive public policies can provide.

Another critical issue is the neglect, in classical models, of the psychosocial conditions influencing entrepreneurial performance. The narrow focus on technical skills and capital overlooks the fact that, in the context of vulnerability, factors such as trust networks, peer solidarity, and emotional support may be more decisive for the survival of a business than formal management competencies [3,10,11]. This conceptual myopia leads to ineffective public and private interventions.

In the realm of public policy, the uncritical adoption of the traditional model has led to the implementation of standardized training and credit programs, based on the assumption that all entrepreneurs share the same starting conditions. However, empirical evidence shows that such initiatives, when disconnected from the intersectional realities of low-income women, tend to reinforce inequalities, disproportionately benefiting those who already have higher levels of education, established networks, and some accumulated capital [2]. Recent evidence from Brazil reinforces this critique, Melo et al. (2025) [33] evaluated a financial training intervention with women entrepreneurs and found significant improvements not only in financial knowledge but also in socio-emotional competencies and support networks, underscoring the relevance of training models tailored to intersectional realities.

The indiscriminate application of classical conceptions of entrepreneurship in the Global South thus proves insufficient and, at times, counterproductive. It is necessary to acknowledge that entrepreneurial activity is contextual and anchored in specific dynamics shaped by historical, cultural, and political factors that transcend the scope of traditional definitions. This recognition requires not only methodological adjustments, but also an epistemological revision capable of incorporating the plurality of entrepreneurial forms, including those that prioritize well-being and community cohesion over purely economic scalability.

## 3. Vulnerability, Intersectionality, and the Impacts of the Pandemic

Socioeconomic vulnerability is a structural phenomenon that manifests itself through intersectionality, becoming acute when interwoven with markers such as gender, race/ethnicity, class/income, and migratory status [9,10,11]. This vulnerability is continuous, not temporary, reinforced by historical processes of exclusion and inequality [34]. Across the Global South, these markers combine with factors such as informality, institutional instability, and inadequate public policies, creating a field of constraints that influences not only women’s entry into entrepreneurship, but also their capacity to stay in it [2,3]. The absence of formal support networks and restricted access to credit and technology further exacerbates the fragility of these initiatives. As such, when we examine the highly gendered impacts of the COVID-19 pandemic restrictions, it is clear that lockdowns and broader restrictions did not create new inequalities, but exacerbated existing ones [12,13].

The COVID-19 pandemic constituted a systemic shock, simultaneously affecting supply and demand and disrupting essential supply chains for small businesses [10,16]. Mobility restrictions disrupted customer flows, compromised the acquisition of inputs, and hindered the maintenance of commercial partnerships. For women with caregiving responsibilities, the overlap of domestic and productive demands became unsustainable, leading to physical and emotional exhaustion that directly affected their ability to keep their businesses operating [3,14].

The challenge of vulnerability is highly contextual, thus excluding broad generalizations throughout the Global South [35]. The intersectional lens is essential to reveal these specificities: for instance, in the Latin American context, the historical legacy of colonialism places race as a central determinant of inequality, and black entrepreneurs face systemic discrimination that restricts access to credit and formalization [1,3,4]. In contrast, in many African contexts, the primary barriers are often rooted in deeply entrenched patriarchal structures and institutional fragility, which restrict women’s autonomy, often mediated by other social identifiers such as ethnicity, religion and the rural/urban divide [2,6].

Furthermore, the specific vulnerability of refugee and asylum seekers must be distinguished from voluntary migration, as their lack of legal protection and limited social capital require reliance on informal entrepreneurship, amplifying intersectional barriers such as xenophobia and trauma [2,12,26]. These distinct realities necessitate contextual analysis that move beyond homogeneous categorization, recognizing how specific markers (gender, race, ethnicity legal status) are magnified by local socio-political landscapes.

The impact of this multi-layered crisis extends beyond the economic sphere: issues such as gender-based violence, social isolation, and deterioration of mental health were exacerbated during the pandemic and had profound indirect impacts on women’s entrepreneurial activity [3,10]. This requirement for a holistic response highlights the insufficiency of homogeneous policies. It is therefore evident that post-pandemic recovery strategies must incorporate integrated, context-sensitive instruments that recognize the plurality of realities among women entrepreneurs in the Global South. This involves formulating intersectional policies that integrate gender, race/ethnicity, class/income, and legal migration status as central variables in the design of credit, training, technological support, and social protection programs [2,17]. Only through such an integrated approach will it be possible to build a more inclusive and resilient entrepreneurial ecosystem.

## 4. Mental Health, Public Policies, and Collective Strategies

Survival related challenges became even more important for vulnerable female entrepreneurs in the post-pandemic recovery period. This was because public policies for income generation remained insufficient and were further stretched by the almost total absence of mental health support in most places around the world, but certainly to a more significant extent in many parts of the Global South. However, the business sustainability and personal well-being of low-income women entrepreneurs greatly depend on ensuring that appropriate mental health support is available to them. In times of crisis, such as the COVID-19 pandemic, rising levels of stress, anxiety, and depression have been widely documented among the general population, and take different dimensions when we consider the lives of those balancing multiple work roles and facing financial instability [3,10]. This, as the emotional stress resulting from the combined burden of productive and reproductive labor, directly affects decision-making capacity, creativity, and resilience, all essential competencies for maintaining businesses in adverse environments. In this sense, policies that integrate entrepreneurial support with mental health care function not only as mechanisms for economic inclusion but as vital public health and health promotion interventions for women exposed to chronic stress and social exclusion.

The relationship between mental health and entrepreneurship is further complicated, in many contexts across the global South, by structural gaps in public health systems. Entrepreneurship support programs rarely include components for mental health prevention and care, operating as if the psychological sphere were detached from business management [12]. This omission reflects not only institutional neglect but also an epistemological limitation: the persistence of entrepreneurship models that treat the individual as a self-sufficient unit, disregarding collective and emotional factors. The Brazilian context illustrates these tensions. For example, the Microempreendedor Individual (MEI) scheme, created to formalize small businesses, ensures access to some social security and health benefits through the Unified Health System (SUS). However, coverage does not systematically include mental health services for entrepreneurs, revealing a disconnect between public policy and the actual needs of these women [12,16]. In neighboring Latin American countries, similar initiatives present the same shortfall, prioritizing formalization and credit without integrating psychological well-being measures.

The Pro Mujer initiative represents an important example of integrated microfinance practice by combining group-based lending with preventive health care and psychosocial support for women entrepreneurs in Latin America. Evidence from Pro Mujer Mexico demonstrates that external social ties function as informal risk insurance arrangements and information channels that strengthen repayment performance when they embed valuable financial and non-financial resources [36,37]. Building on this logic, programmes that integrate financial inclusion with health services and psychosocial assistance tend to enhance business resilience, particularly in post-crisis and post-pandemic contexts, where emotional stability, social support and network embeddedness are central to enterprise survival. Rwanda offers another case of best practice, as the government opted to go beyond credit schemes and linked its entrepreneurial policies with community health programs, including counseling and collective support groups, demonstrating the benefits of integrating economic inclusion with mental health care [38].

The lack of integrated policies is also evident in migratory contexts. Refugee or migrant women who engage in entrepreneurial activity face additional barriers to accessing health services, due to legal restrictions or linguistic and cultural barriers [2,12]. In such cases, social support becomes crucial, functioning as both a protective network and an informal channel for circulating information about rights and opportunities. However, the effectiveness of these networks is highly dependent on the organizational capacity of the communities and the presence of intermediary social actors. In this regard, collective strategies emerge as powerful alternatives for mitigating the effects of vulnerability and strengthening entrepreneurial resilience. Women’s collectives, cooperatives, and community associations by sharing resources, knowledge, and infrastructure reduce individual dependency and expand the reach of initiatives [10,17], such arrangements also create spaces for emotional support, which are essential to cope with management pressures in high-uncertainty environments.

Beyond functioning as recipients of social support, refugee and immigrant women actively participate in shaping collective spaces and community-based infrastructures that sustain entrepreneurial resilience [10,12]. In many contexts, small-scale entrepreneurial settings operate not only as economic units but also as sites of collective organization, information exchange, and mutual care [12,17]. These spaces enable the circulation of knowledge about rights, markets, and opportunities, while simultaneously fostering emotional support and collective coping strategies [10,17]. By transforming everyday entrepreneurial environments into arenas of social interaction and coordination, women contribute to the construction of collective resilience that links individual survival strategies to broader community responses [12]. This collective dimension reinforces entrepreneurial sustainability while mitigating isolation, psychosocial stress, and uncertainty in contexts marked by legal precarity and social exclusion [10,17]. Beyond government action, the inclusion of diverse actors is crucial for building an entrepreneurial ecosystem that encompasses society, universities, and public, private, and non-governmental institutions.

In Brazil, a example is the Instituto Rede Mulher Empreendedora (IRME), which has been recognized for promoting the economic autonomy of women in situations of vulnerability through initiatives focused on entrepreneurship, employability and community strengthening. The Institute directs its efforts particularly towards those who face deeper barriers in accessing opportunities—such as women experiencing violence, residents of informal settlements and low-income communities, former convicts, refugees, Black women, trans women, women aged 50+, among others whose trajectories are marked by social and economic vulnerabilities [39]. The qualitative evidence presented by Carlini (2022) [40] demonstrates that the RME operates as a structured national support network, combining voluntary leadership, localized action, mentoring, technical training and collective learning spaces, which are perceived by its ambassadors as generating measurable socioeconomic impact, particularly in terms of knowledge acquisition, business development, networking and self-confidence. By articulating capacity-building, peer support and regional mobilization, IRME strengthens women’s economic autonomy not only at the individual level but also through community-based network effects that enhance collective empowerment. Another significant initiative, still in the Brazilian context, is the Fundo Agbara, aimed at Black women. Created in 2020, the fund has already mobilized more than BRL 1 million to promote income generation and productive inclusion, strengthen racial and gender empowerment, stimulate entrepreneurship and the creative economy, foster a culture of giving and support community development [41]. Additionally, private initiatives in partnership with international organizations, such as the Itaú Mulher Empreendedora + DIVER.SSA program, have strengthened Black and Indigenous women’s entrepreneurship by providing technical and emotional support, resulting in a significant expansion of small businesses [42,43].

Furthermore, the Aliança para Gênero e Empoderamento Feminino no Financiamento Internacional (Alliance for Gender and Women’s Empowerment in International Financing), established in 2024, enhances strategies to increase women’s participation in projects funded with external resources, through multilateral programs, such as WeForLAC, funded by the Inter-American Development Bank (IDB), to provide access to credit, mentoring networks, and markets for women entrepreneurs in countries such as Honduras, El Salvador, Guatemala, Mexico, Colombia, Brazil, Peru, Ecuador, and the Dominican Republic [44,45].

Evidence from Latin America demonstrates that collective interventions are more effective than individual solutions. Microcredit programs combined with training and collective mentorship have positively impacted both entrepreneurial skill development and the reduction of women’s emotional burden [12]. This was also the case in Africa where Bayisenge et al. (2020) [46], based on a sample of 195 women entrepreneurs in Rwanda, identified that their businesses play a crucial role in creating jobs, promoting gender equality and reducing poverty. The authors emphasize that government and non-governmental support is essential to strengthen micro and small enterprises, particularly in rural areas, thereby fostering economic growth, social inclusion, and increased household income. In addition the government in Rwanda has played a central role in developing programs aimed at women’s economic inclusion through tax incentives and business management training.

In Asia, countries such as Bangladesh also stand out for microfinance programs such as Grameen Bank, which have increased women’s access to credit and economic autonomy [47]. However, studies indicate that without parallel social protection and mental health policies, these advances can be limited or even generate new pressures on women, who accumulate both productive and reproductive responsibilities [48]. This demonstrate the importance of community-based strategies, such as the La Cortesana initiative in Colombia connecting informal sewing workshops with major brands and generating dignified and sustainable work for hundreds of women [49]. Similarly the Cooperativa Las Productivas in the Dominican Republic, supported by the Fundación Microfinanzas (BBVA), expanded production and transformed cocoa into a source of local development [50].

Thus, strengthening the entrepreneurship of low-income women in the post-pandemic period requires a new paradigm that explicitly integrates mental health, inclusive public policies, and collective support strategies. This integration, grounded in an intersectional vision, not only addresses immediate needs but also lays the foundations for more equitable, resilient, and sustainable development in the Global South. Building this ecosystem depends on coordinated action between government, civil society, universities, and private institutions, recognizing that entrepreneurship, under these conditions, is inseparable from the social and emotional structures that sustain it and the networks that protect it.

## 5. Labor Rights and Public Policies

Natividade (2009) [51] highlights autonomy and strong decision-making power as central characteristics of Brazilian women in the entrepreneurial sphere, noting that they pursue opportunities that, in their view, provide greater financial security. However, these entrepreneurs also face significant barriers, including low levels of education, financial constraints, and excessive bureaucracy, challenges that are deeply rooted in the gender inequalities they experience. Understanding how public policies affect the strengthening of women’s entrepreneurship in this context is therefore essential.

Across Latin America, women’s entrepreneurship frequently arises as a strategy to navigate structural inequalities and limited formal employment. For many women, establishing a business is less a career choice than a means of survival while balancing multiple responsibilities, including childcare and family care, especially for low-income women [52]. This underscores the need for public policies that not only improve access to credit and training but also integrate psychosocial support, as the cumulative emotional burden can generate elevated stress and anxiety [53].

In Africa, women’s entrepreneurialism also emerges as a survival strategy in the face of structural inequalities and the scarcity of formal employment. The continent has the highest rate of entrepreneurialism worldwide and is the only one in which women represent the majority of entrepreneurs [54]. However, African women entrepreneurs face significant obstacles, such as cultural norms, societal expectations, and historical biases that continue to restrict women’s opportunities, generating barriers such as limited mobility, restricted access to finance, and traditional roles that discourage participation in non-traditional sectors [55].

Although microfinance programs are considered relevant alternatives for economic inclusion, studies show that, in contexts such as Lagos, Nigeria, strict repayment requirements provoke high levels of psychological distress and stress among women traders [56]. In contrast, innovative community-based interventions have sought to address this care gap.

At an empirical level, research highlights the complex relationship between women’s entrepreneurship, autonomy, and mental health. In Burkina Faso, women with greater household decision-making power and self-efficacy showed lower levels of anxiety and depression, although access to credit paradoxically appeared to be associated with increased depressive symptoms [57]. In Ghana, Essuman et al. (2024) [58] demonstrated that business resilience, subjective well-being, and social cohesion are interconnected and contribute to the sustainability of women-led enterprises. Malanga and Banda (2021) [59] found that the use of information and communication technologies (ICTs) by rural women entrepreneurs strengthened their financial, social and informational resources, thus promoting autonomy and improving quality of life.

Both Essuman et al. (2024) [58] and Malanga and Banda (2021) [59] highlight that public policies aimed at women’s entrepreneurship must be integrated, combining economic, social, and psychological dimensions. Beyond expanding access to credit and encouraging formalization, it is essential to recognize that mental health and subjective well-being are fundamental pillars for the resilience and continuity of women’s entrepreneurial trajectories as state support is often limited, and entrepreneurial resilience depends on hybrid approaches that combine microcredit, technical training, and community support [12]. Thus, ignoring the mental health of entrepreneurs risks reinforcing existing vulnerabilities rather than fostering sustainable empowerment [60].

This was dramatically exacerbated by the COVID-19 pandemic across Latin America and Africa, reiterating the imperative for a new paradigm moving beyond promoting simple economic inclusion and formalization and requiring the explicit integration of labor rights and legal guaranties with psychosocial support and collective strategies. This integration, grounded in intersectional decision-making considers gender, race/ethnicity, and class/income, as crucial not only for the economic sustainability and the resilience of women’s ventures, but also for their mental health and subjective well-being, emphasizing that the success of sustainable entrepreneurship in the Global South is inseparable from the social, legal, and emotional structures that are put in place to protect and sustain it.

## 6. Conclusions

This paper has argued that the entrepreneurialism of low-income women in the Global South cannot be analyzed through reductionist lenses or conventional economic models. The intersection of gender, race/ethnicity, class/income, and migratory status reveals structurally constrained trajectories that challenge homogeneous conceptions of entrepreneurship and the assumption of full individual agency.

The post-pandemic context has further exposed these inequalities, confirming the health crisis as a catalyst for exacerbating pre-existing vulnerabilities, while also fostering collective strategies and community-based innovations as mechanisms of resistance and reconstruction. However, the sustainability of such initiatives depends on public policies capable of moving beyond neutrality by incorporating intersectional variables as central planning criteria.

In this regard, the integration of mental health, technical training, digital inclusion, and financial support emerges as a necessary and coordinated condition for strengthening women’s entrepreneurship. Without systemic coordination, policy interventions risk reproducing structural inequalities, undermining business sustainability and perpetuating cycles of social and economic exclusion.

From a theoretical standpoint, this paper contributes to advancing entrepreneurship studies by framing entrepreneurial activity as socially embedded and shaped by power structures, institutional arrangements, and health determinants. This perspective bridges entrepreneurship and public health, positioning economic agency as inseparable from psychosocial well-being.

For policy and practice, the findings highlight the need for intersectional program design, institutional capacity-building, and monitoring frameworks that integrate social, economic, and health indicators. Such an approach strengthens entrepreneurial ecosystems while expanding social protection and collective resilience.

Future research should prioritize mixed methods approaches that combine economic impact data with analysis of lived experience, as well as comparative studies across Global South contexts to examine how institutional arrangements shape women’s entrepreneurial trajectories. Greater attention should also be given to the role of formal and informal support networks and to the relationship between public policy and mental health within entrepreneurial ecosystems.

Ultimately, supporting women’s entrepreneurship in vulnerable settings requires moving beyond survivalist narratives toward structural transformation. When supported by intersectional and integrated policies, entrepreneurship operates not only as an economic strategy but also as a pathway to health promotion, psychosocial resilience, and social justice, reinforcing the Global South as a central site of knowledge production.

## Data Availability

Not applicable.

## Abbreviations

The following abbreviations are used in this manuscript:

MEI	Individual Microentrepreneur Scheme
SUS	Unified Health System
GEM	Global Entrepreneurship Monitor
UNDP	United Nations Development Programme
IRME	Women Entrepreneur Network Institute
ICTs	Information and Communication Technologies
AFD	French Development Agency
IDB	Inter-American Development Bank
CAF	Development Bank of Latin America and the Caribbean
IFC	International Finance Corporation
BBVA	Fundación Microfinanzas
